# Prognostic Nutritional Index in Hepatocellular Carcinoma Patients With Hepatitis B Following US-Guided Percutaneous Microwave Ablation: A Retrospective Study With 1,047 Patients

**DOI:** 10.3389/fsurg.2022.878737

**Published:** 2022-06-29

**Authors:** Yaxi Wang, Xin Li, Jie Yu, ZhiGang Cheng, Qidi Hou, Ping Liang

**Affiliations:** Department of Interventional Ultrasound, Chinese PLA General Hospital, Beijing, China

**Keywords:** hepatocellular carcinoma, hepatitis B virus, prognostic nutritional index, microwave ablation, overall survival

## Abstract

**Objective:**

Several studies have revealed that the prognostic nutritional index (PNI) was associated with survival in several cancers. However, the prognostic value of PNI in hepatocellular carcinoma (HCC) patients following ultrasound-guided percutaneous microwave ablation (US-PMWA) remains unknown, especially in patients with hepatitis B virus (HBV) infection. Therefore, the present study aimed to evaluate the potential prognostic value of PNI in these patients.

**Materials:**

The medical records of 1,047 HCC patients with HBV infection following US-PMWA were retrospectively reviewed. The association between preoperative PNI and overall survival (OS), as well as other clinical characteristics of HCC, were analyzed using the Kaplan–Meier plot, log-rank test, multi-parameter Cox proportional hazards model, restricted cubic spline (RCS), and time-dependent receiver operating characteristic (ROC) curve analyses.

**Results:**

Patients with a preoperative PNI more than 45 were verified to have better OS than patients with a PNI less than 45. In the multi-parameter Cox proportional hazards models, the log-transformed PNI was verified as an independent prognostic factor for OS. The result of the RCS analysis revealed that there was a nearly linear relationship between PNI and OS. The area under the time-dependent ROC curve for PNI in predicting OS was 0.56, which is relatively stable.

**Conclusion:**

Preoperative PNI represents a convenient, noninvasive, and independent prognostic indicator in HCC patients with HBV infection following US-PMWA.

## Introduction

Hepatocellular carcinoma (HCC) is listed as the sixth most common cancer and the fourth leading cause of cancer-related death worldwide (1). The 5-year overall survival (OS) of HCC is approximately 18%, which ranks it as the second most lethal tumor after pancreatic cancer (2). In China, HCC has the fourth highest incidence rate and the third highest mortality rate, with a 5-year OS of 12% (3). According to the European Association for the Study of the Liver (EASL) and American Association for the Study of Liver Diseases (AASLD) guidelines, the Barcelona Clinic Liver Cancer (BCLC) staging system is the preferred model for HCC treatment allocation and prognostication (4, 5). Liver resection, liver transplantation, and ablation are recommended as the first-line radical treatments for HCC. Importantly, ablation is recommended for the patients with BCLC stage 0 and A, who are not candidates for surgical intervention (6, 7). Ablation is mainly performed under the guidance of imaging (US, CT, MRI, X-ray), and causes tumor necrosis by inducing a high intratumoral temperature (2). Among the thermal ablation techniques, microwave ablation (MWA) achieves higher thermal efficiency, resulting in a larger ablation zone with shorter ablation and anesthesia times, and has relatively low ablation-related complications (8, 9). Although radical treatments including liver transplantation, resection, and ablation have made some progress in recent years, the 5-year metastasis and recurrence rate after treatment is still as high as 70% (10). Meanwhile, the therapeutic strategy and prognosis of HCC mainly depends on the clinical stage of the tumor and the hepatic functional reserve (11). Therefore, it is imperative to explore an efficient and convenient system for clinicians to predict prognosis and optimize individual treatment strategies for HCC patients.

HCC usually occurs with hepatitis infection (including hepatitis B virus [HBV], hepatitis C virus [HCV]), alcohol abuse, and non-alcoholic fatty liver disease (12, 13). Chronic inflammation and cirrhosis underline most HCC cases and are both independent risk factors for HCC, and lead to impaired hepatic functional reserve and hypersplenism (14). Hypoproteinemia is a common index of the poor hepatic functional reserve, and thrombopenia is a common symptom of hypersplenism. Hypoproteinemia and thrombopenia often occur in HCC patients with severe liver cirrhosis and are closely related to HCC prognosis. Recently, the statuses of body immunity and nutrition were shown to be closely associated with the prognosis of various cancers. Many indicators containing immunity and nutritional indexes have been verified as predictive factors for the prognosis of many cancers. Among these indicators, the prognostic nutritional index (PNI) was proposed first in 1980 to evaluate its prognostic value for patients who underwent gastrointestinal surgery (15), and was calculated based on serum albumin (ALB) levels and peripheral blood lymphocyte count. The PNI has been used to assess the immunonutritional status of cancer patients and has been verified as a useful prognostic indicator in many cancers (16, 17). In China, HBV infection is one of the most important risk factors for HCC and is responsible for approximately 80% of virus-associated HCC patients (18). Moreover, antiviral therapy may reduce the risk of tumor recurrence and improve OS among HCC patients with HBV infection after ablation (19). Therefore, the value of PNI in HCC patients with an HBV infection following ablation should be explored, and might provide reasonable and effective treatments to improve long-term prognosis.

Until now, some research has indicated that the preoperative PNI was an independent prognostic factor for relapse-free survival (RFS) and OS in HCC patients who received hepatectomy (20). However, there are no studies on the prognostic value of PNI in HCC patients with HBV infection following ablation. Therefore, the main purpose of the present study was to evaluate the prognostic value of preoperative PNI in HCC patients with HBV infection who underwent ultrasound-guided percutaneous microwave ablation (US-PMWA) for radical elimination.

## Methods and Materials

### Study Population and Design

This is a retrospective study performed at a single center. The Ethics Committee of the Chinese PLA General Hospital (Beijing, China) approved the protocol (S2020-432-01). The study was conducted as per the Declaration of Helsinki. A total of 1,563 treatment-naive HCC patients treated by US-PMWA were enrolled between Jan 2002 and Dec 2017. Written informed consent was obtained from all patients. The diagnoses of HCC and cirrhosis were confirmed either by histopathological diagnosis using a prior biopsy or through imaging [including contrast-enhanced ultrasound (US), contrast-enhanced computed tomography (CT) and/or magnetic resonance imaging (MRI)], based on the Guidelines for the Diagnosis and Treatment of Primary Liver Cancer of China.

The inclusion criteria were as follows: (1) Eastern Cooperative Oncology Group performance scores (ECOG) ≤2; (2) single tumor with maximum diameter ≤5 cm; (3) patients with hepatitis B who were positive for hepatitis B soluble antigen (HBsAg); (4) Child-Pugh A or B; (5) absence of vascular invasion or extrahepatic metastases; (6) serum total bilirubin (TBI) level <50 mol/L; (7) ALB level >25 g/L; (8) platelet count > 50 × 10^9^/L and prothrombin activity >50%; (9) final diagnosis was based on the pathologic findings; (10) without a history of other malignancies; and (11) refused to receive liver resection or transplantation. The exclusion criteria were as follows: (1) incomplete clinical data; (2) lost to follow-up within 3 months after ablation; and (3) serious heart, lung, and renal dysfunction, and severe active infection.

### Data Collection

The clinical parameters collected were as follows: (1) patient characteristics (age, sex, pathological diagnosis, cirrhosis, Child-Pugh degree and ascites); (2) tumor characteristics (tumor maximum diameter (D), number and location); (3) preoperative data [alpha-fetoprotein (AFP), alanine transaminase (ALT), aspartate aminotransferase (AST), ALB, TBI, direct bilirubin (DBI), total protein, cholinesterase, gamma-glutamyl transpeptidase (GGT), serum creatinine, hemoglobin, platelet, prothrombin time (PT) and international normalized ratio (INR)]; and (4) survival. PNI was calculated as serum albumin (ALB) (g/L) + 5 × total lymphocyte count (10^9^/L). The PNI cut-off value was defined as 45 according to a previous report (20).

### US-PMWA Procedure

The microwave system with two cooled-shaft and frequencies of 2450 MHz was used (KY-2000, Kangyou Medical, Nanjing, China). The needle antenna with a diameter of 1.9 mm and an 18 cm shaft could be easily visualized in images. A narrow radiating segment of 3 mm was embedded on the shaft, 11 or 22 mm away from the tip. A real-time thermal monitoring system was equipped in the microwave machine to continuously measure the temperature during the ablation process. The diameter of the thermal monitoring needle was 0.8 mm.

The US-guided biopsy was collected immediately before ablation by an automatic biopsy gun with an 18 ga cutting needle. Subsequently, the antennas were percutaneously inserted into the tumor and placed at the designated location. If the tumor had a maximum diameter ≤1.7 cm, one antenna was applied. Two antennae were used simultaneously with repeated insertion for tumors with a maximum diameter >1.7 cm. During the ablation, a power output of 50–60 W was applied. Occasionally, a real-time thermal monitoring needle was placed at the tumor margin or important organs adjacent to the tumor for temperature monitoring. After all insertions, intravenous anesthesia was administered. When the heat-generated hyperechoic water vapor covered the entire tumor as visualized by the US, the ablation was considered successful. If the tumor was located in a relatively risky area and was >3 cm, the 3D visualization operative planning system, artificial pleural effusion and ascites were also applied (21, 22).

### Follow-up

Contrast-enhanced ultrasound (CEUS) and contrast-enhanced CT/MRI were performed to assess the treatment effect at 3 days after ablation. If irregular peripheral enhancement in a scattered, nodular, or eccentric pattern occurred, additional ablation was performed. Otherwise, routine contrast-enhanced images and serum tumor markers were reviewed after 1 and 3 months, and then every 6 months. Chest radiography was applied every 6 months and bone scintigraphy was applied yearly to check for extrahepatic metastasis. Thereafter, they were followed up by telephone. The endpoints of this study were death or study termination. OS was considered as the interval from ablation completion to death or the date of the last or most recent follow-up visit. Complications were recorded according to the Society of Interventional Radiology classification system for complications by outcome (23, 24). If recurrence and/or metastasis were detected, proper treatments (repeat ablation, transarterial chemoembolization (TACE), targeted therapy, immunotherapy and supportive treatment) were administered.

### Statistical Analysis

The Kolmogorov–Smirnov test was used on continuous variables to assess for normal distribution. Normally distributed data were compared using an unpaired t-test. Otherwise, the Mann–Whitney test was used. Categorical variables were compared by Chi-square or Fisher's exact test (25). OS was graphically depicted by the Kaplan–Meier curve and compared using the log-rank test. Multivariate Cox regression models were used to analyze the hazard ratios (HRs) of the baseline characteristics for OS. The variable selection method used in the multivariate analysis was “enter.” Constrained cubic spline and time-dependent receiver operating characteristic (ROC) curves were performed to assess the prognostic value of PNI. R-version 4.1.1 was used for the data analysis. A *P* value less than 0.05 was considered statistically significant.

## Results

### Patient Characteristics

**Figure 1:**
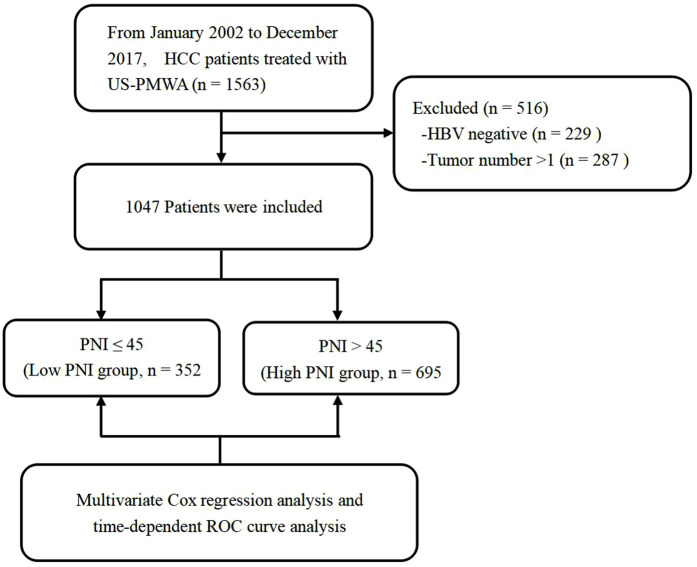
Flow chart of patient selection and study design.

According to the inclusion and exclusion criteria, 516 patients were excluded from the study. Details regarding patient selection and study design are shown in Figure 1. A total of 1,047 HCC patients with HBV infection who received US-PMWA from Jan 2002 to Dec 2017 at our center were included for the final analysis. The clinicopathological characteristics of the 1,047 HCC patients were shown in Table 1. The present study included 857 men (82%) and 190 females (18%), with a median age of 58 years. The patients were divided into the low PNI group (*n* = 352) and the high PNI group (*n* = 695), with an insignificant difference in sex between the two groups (*P* = 0. 143). The median age in the low PNI group (60 years) was significantly older than that in the high PNI group (57 years) (*P* < 0.001). The comorbidity of diabetes insignificantly differed between the low PNI group (138, 40%) and high PNI group (230, 33%) (*P* = 0.059). The diameter of tumors in the low PNI group (D = 2.6) was significantly larger than that in the high PNI group (D = 2.4) (*P* = 0.003). Although all patients were infected with the hepatitis B virus, a significant difference in liver cirrhosis was detected with 342 (97%) in the low PNI group and 653 (94%) in the high PNI group (*P* = 0.036). There were 28 (8%) patients with Child-Pugh B in the low PNI group, and 16 (2%) patients with Child-Pugh B in the high PNI group, which was a significant difference (*P* < 0.001). The incidence of ascites was also significantly higher in the low PNI group (46 patients, 13%) compared to the high PNI group (17 patients, 2%) (*P* < 0.001). There was no significant difference in the lesion location between the low and high PNI groups (*P *= 0.056). As for the laboratory examination, significant differences between the two groups were detected in most indexes, including AST, ALT, AFP, GGT, total protein, ALB, TBI, DBI, creatinine, cholinesterase, platelet, leukocyte, neutrophil, lymphocyte, hemoglobin, PT and INR (*P *< 0.05). The results indicated that patients in the low PNI group experienced poor immunonutritional status compared to the high PNI group. No severe complication related to MVA was found. Mild to moderate complications included fever, ascites, and pleural fluid, which were resolved with drug-based and supportive treatments. Regarding ascites, significant differences were detected between the two groups (*P* = 0.001), while no significant differences in fever and pleural fluid were found between the low and high PNI groups (*P* = 0.402, *P* = 0.185, respectively).

**Table 1:** The relationship between clinicopathological factors and PNI in HCC patients with HBV infection following US-PMWA.

Variables	Total (*n* = 1047)	PNI ≤ 45 (*n* = 352)	PNI > 45 (*n* = 695)	*P* value
Gender, *n* (%)				0.143
Female	190 (18)	73 (21)	117 (17)	
Male	857 (82)	279 (79)	578 (83)	
Age, years	58 (50, 66)	60 (53, 67)	57 (49, 65)	<0.001
Diabetes, *n* (%)				0.059
No	679 (65)	214 (61)	465 (67)	
Yes	368 (35)	138 (39)	230 (33)	
Diameters, cm	2.4 (1.8, 3.2)	2.6 (1.9, 3.3)	2.4 (1.8, 3.1)	0.003
Liver cirrhosis, *n* (%)				0.036
No	52 (5)	10 (3)	42 (6)	
Yes	995 (95)	342 (97)	653 (94)	
Child-Pugh, *n* (%)				<0.001
A	1,003 (96)	324 (92)	679 (98)	
B	44 (4)	28 (8)	16 (2)	
Ascites, *n* (%)				<0.001
No	984 (94)	306 (87)	678 (98)	
Yes	63 (6)	46 (13)	17 (2)	
Near organ, *n* (%)				0.056
No	506 (48)	155 (44)	351 (51)	
Yes	541 (52)	197 (56)	344 (49)	
ALT, U/l	24.9 (17.9, 36.1)	26.9 (19.5, 40.2)	24.3 (17.7, 34.5)	0.015
AST, U/l	25.0 (20.0, 34.8)	29.4 (22.4, 41.3)	23.4 (19.2, 30.3)	<0.001
GGT, U/l	38.9 (26.1, 70.1)	47.3 (29.5, 81.1)	36.5 (25.0, 62.1)	<0.001
AFP, ng/mL	12.29 (3.53, 77.76)	16.55 (4.22, 88.70)	9.93 (3.29, 72.11)	0.042
TP, g/l	67.0 (63.5, 71.0)	64.5 (60.9, 67.8)	68.0 (65.3, 72.0)	<0.001
ALB, g/l	40.1 (37.2, 42.8)	36.2 (33.0, 38.2)	41.9 (39.7, 43.7)	<0.001
TBIL, μmol/l	14.9 (11, 20.6)	18.5 (12.9, 27.5)	13.7 (10.3, 17.8)	<0.001
DBIL, μmol/l	5.1 (3.6, 7.5)	7.00 (4.9, 10.4)	4.5 (3.3, 6.1)	<0.001
Creatinine, μmol/l	70.3 (62, 79.9)	67.3 (58.1, 76.3)	72.1 (63.4, 81.4)	<0.001
Cholinesterase, U/l	5978 ± 1837	4771 ± 1532	6589 ± 1669	<0.001
Hemoglobin, g/l	138 (126, 148)	128 (116, 138)	143 (132, 152)	<0.001
Platelet, 10^9^/l	112 (76, 154)	72 (49, 102)	131 (99, 166)	<0.001
Leukocyte, 10^9^/l	4.42 (3.42, 5.43)	3.32 (2.58, 4.36)	4.85 (4.04, 5.76)	<0.001
Neutrophil, %	0.56 (0.50, 0.62)	0.58 (0.52, 0.65)	0.55 (0.49, 0.61)	<0.001
Lymphocyte, %	0.33 (0.27, 0.39)	0.28 (0.23, 0.35)	0.35 (0.29, 0.40)	<0.001
PT, second	14.2 (13.5, 15.1)	15.2 (14.2, 16.7)	13.9 (13.3, 14.5)	<0.001
INR	1.10 (1.05, 1.19)	1.20 (1.11, 1.33)	1.08 (1.02, 1.13)	<0.001
Complications				
Fever (Y/N)	547/500	177/175	370/325	0.402
Ascitic fluid (Y/N)	6/1041	6/346	0/695	0.001
Pleural fluid (Y/N)	20/1027	10/342	10/685	0.185
Follow up time, day	928 (546, 1467)	862 (529, 1381)	953 (556, 1516)	0.067
Status, *n* (%)				<0.001
Alive	614 (59)	180 (51)	434 (62)	
Died	433 (41)	172 (49)	261 (38)	

*PNI, prognostic nutritional index; HCC, hepatocellular carcinoma; US-PMWA, ultrasound-guided percutanous microwave ablation; HBV, hepatitis B virus; AFP, alpha-fetoprotein; ALT, alanine transaminase; AST, aspartate aminotransferase; GGT, gamma-glutamyl transpeptidase; PT, prothrombin time; INR, international normalized ratio; TP: total protein; ALB, albumin; TBIL, total bilirubin; DBIL, direct bilirubin.*

### The Relationship of PNI and Long-Term Outcomes

**Figure 2:**
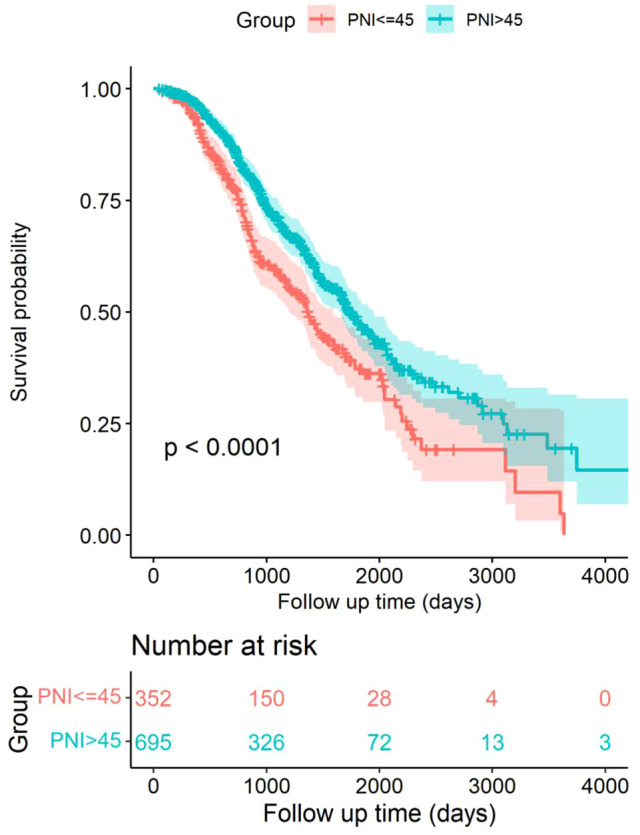
Kaplan–Meier survival curves comparing HCC patients within Milan criteria after US-PMWA with low PNI and high PNI groups.

During the follow-up period 928 days, a total of 172 patients (49%) died in the low PNI group and 261 patients (38%) died in the high PNI group (*P* < 0.001). The Kaplan–Meier survival curves revealed that patients in the low PNI group had significantly better OS rates than the high PNI group (*P *<  0.001) (Figure 2). Further, the results of multivariate Cox regression models were used to analyze the hazard ratios (HRs) of the baseline characteristics of OS. In Model 1, log-transformed PNI (HR = 0.14, 95% confidence interval (CI): 0.06–0.32, *P *< 0.001), tumor diameter (HR = 1.25, 95% CI: 1.13–1.37, *P* < 0.001), fever (HR = 0.77, 95% CI: 0.64–0.94, *P* = 0.009) and tumor adjacent organs (HR = 0.77, 95% CI: 0.64–0.94, *P* = 0.010) were independent risk factor for OS in HCC patients. In Model 2, similar results were detected. Log-transformed PNI (HR = 0.13, 95% CI: 0.06–0.30, *P *< 0.001), tumor diameter (HR = 1.25, 95% CI: 1.14–1.38, *P* < 0.001), fever (HR = 0.77, 95% CI: 0.63–0.93, *P *= 0.008) and tumor adjacent organs (HR = 0.78, 95% CI: 0.64–0.95, *P* = 0.012) were independent risk factor for OS in HCC patients. Therefore, the results indicated that PNI was an independent risk factor for OS in HCC patients (Table 2).

**Table 2:** Multivariate analysis of prognostic factors of OS in HCC patients with HBV infection following US-PMWA by two models.

	HR (95%CI)	*P*
**Model 1**		
Log (PNI)	0.14 (0.06–0.32)	<0.001
Age	1.07 (0.93–1.20)	0.216
Cirrhosis	0.97 (0.57–1.65)	0.922
Diabetes	0.95 (0.74–1.22)	0.679
Ascites	1.21 (0.85–1.74)	0.293
AFP (<35)	0.87 (0.71–1.06)	0.166
Creatinine	0.87 (0.55–1.37)	0.548
TBIL	1.03 (0.83–1.28)	0.782
Platelet (<100)	0.74 (0.59–0.93)	0.009
Tumor diameter	1.25 (1.13–1.37)	<0.001
Adjacent organs	0.77 (0.64–0.94)	0.010
AST	0.95 (0.77–1.17)	0.631
GGT	1.14 (0.99–1.30)	0.060
Fever	0.77 (0.64–0.94)	0.009
Ascitic fluid	0.73 (0.22–2.36)	0.594
Pleural effusion	1.10 (0.56–2.17)	0.778
**Model 2**		
Log (PNI)	0.13 (0.06–0.30)	<0.001
Age	1.07 (0.95–1.20)	0.254
Cirrhosis	1.01 (0.60–1.70)	0.979
Diabetes	0.96 (0.75–1.23)	0.738
AFP < 35 ng/mL	0.87 (0.71–1.06)	0.155
Creatinine	0.89 (0.56–1.40)	0.608
Child-Pugh	1.32 (0.90–1.96)	0.159
Platelet (<100)	0.75 (0.60–0.93)	0.009
Tumor diameter	1.25 (1.14–1.38)	<0.001
Adjacent organs	0.78 (0.64–0.95)	0.012
AST	0.94 (0.77–1.16)	0.595
GGT	1.13 (0.99–1.29)	0.066
Fever	0.77 (0.63–0.93)	0.008
Ascitic fluid	0.74 (0.23–2.40)	0.619
Pleural effusion	1.11 (0.56–2.18)	0.764

*PNI, prognostic nutritional index; HCC, hepatocellular carcinoma; US-PMWA, ultrasound-guided percutanous microwave ablation; HBV, hepatitis B virus; HR, hazard ratio; CI, confidence interval; AFP, alpha-fetoprotein; AST, aspartate aminotransferase; GGT, gamma-glutamyl transpeptidase; TBIL, total bilirubin*.

**Figure 3:**
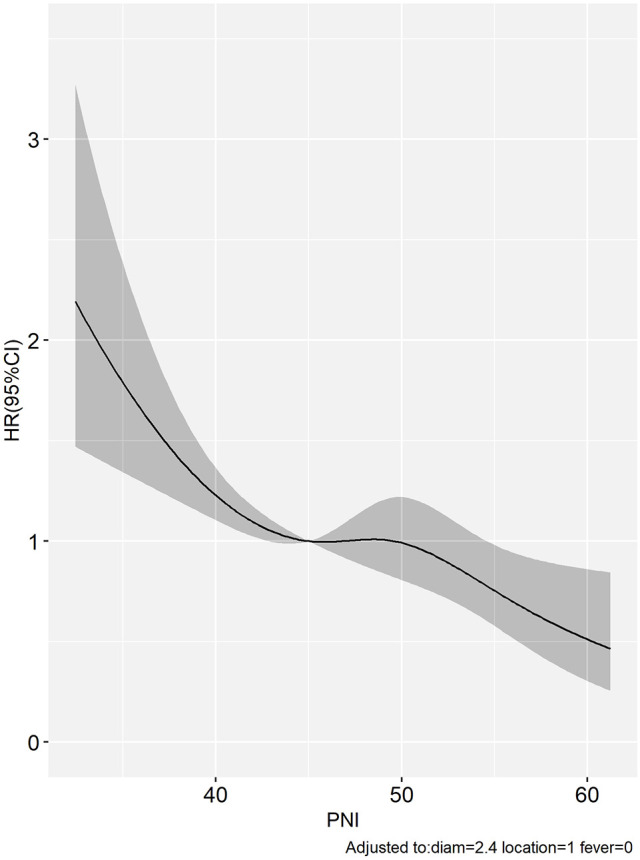
The limited cubic spline showed that a significant negative correlation between PNI and OS in HCC patients within Milan criteria after US-PMWA.

Hazard ratios and 95% confidence intervals for preoperative PNI values were presented with regression dilution bias, and restricted cubic splines (RCS) were reported without this correction. Correction for regression dilution bias was performed using a non-parametric method to correct for underestimation caused by random measurements and long-term fluctuations (26, 27). Therefore, the results of the RCS analysis in the study revealed that there was a significant negative correlation between preoperative PNI and OS (Figure 3).

**Figure 4:**
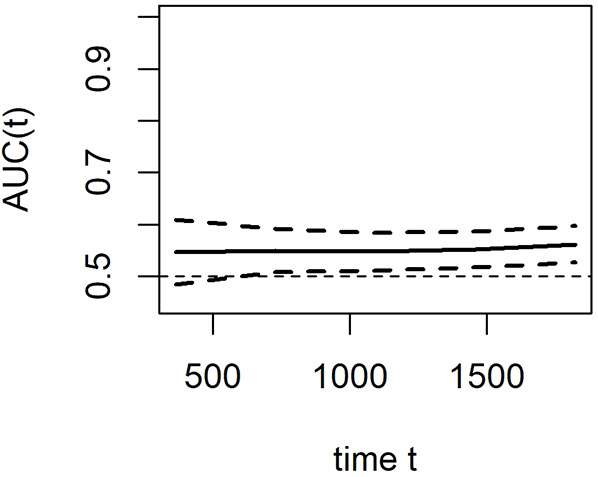
Time-dependent ROC curves indicated the prognostic value of PNI for OS in HCC patients within Milan criteria after US-PMWA.

In the standard ROC curve analysis, the disease status of the individual was defined based on whether the marker value exceeded a threshold value, which was assumed to be fixed during the entire study period. However, the study period commonly had a long follow-up, and individuals who were disease-free earlier may develop the disease later. On the contrary, the disease status of the individual is observed and updated at each time point in the time-dependent ROC curve analysis. With additional information of disease onset at any particular time for each individual, a ROC curve can be constructed at several time points and the marker's predictive ability can be compared (28). Therefore, the time-dependent ROC curve is verified as an efficient method for assessing the predictive value of a candidate indicator given the positive events at certain time points. In this study, the results of the time-dependent ROC analysis revealed that the area under the curve of PNI with a cut-off of 45 was approximately 0.56 for predicting OS (Figure 4). Although the predictive ability for prognosis is weak, it is relatively stable, which also indicates that PNI is closely related to OS of HCC after US-PMWA.

## Discussion

In the past decades, increasing evidence has indicated that immunity and nutritional status play a vital role in tumorigenesis, development, progression, and prognosis of various malignancies. Recently, a low PNI value was considered a valuable prognostic factor, and has been shown to be correlated with poor survival in many cancers (29–31). Most studies emphasized a preoperative PNI cut-off value of 45 *via* ROC curve analysis (29). HCC commonly occurs on the background of liver cirrhosis, and can subsequently lead to hypersplenism. On the contrary, sever liver cirrhosis and hypersplenism are closely correlated with a decrease in albumin and lymphocyte counts. Therefore, PNI might be a promising and valuable prognostic factor for HCC after treatment. Until now, several studies have reported that PNI was an independent predictive factor for survival and closely associated with AFP, tumor size and TNM stage in HCC patients who underwent resection (31–33). Local ablation is used widely in the clinic, and it has several advantages including destroying tumors, decreasing tumor burden, increasing released tumor-related antigen, and activating antitumor immunity (34, 35). Moreover, compared with liver resection, local ablation could protect hepatic function by minimizing the destruction of normal liver tissue. To the best of our knowledge, there have not been any studies performed to evaluate the predictive value of PNI after local ablation in HCC patients with HBV infection.

In the current study, the results demonstrated that preoperative PNI is an independent predictive indicator for OS in HCC patients with HBV infection following US-PMWA. A preoperative PNI value <45 in HCC patients is correlated with a poor prognosis. This result is consistent with many previous studies, which explore the prognostic ability of preoperative PNI in various other cancers (29, 30, 36, 37). For the laboratory examination of HCC patient features, the levels of total protein, ALB and CHE in patients with a low PNI were significantly lower compared to patients with a high PNI. Additionally, the incidence of ascites in patients with a low PNI was significantly higher compared to patients with a high PNI. Therefore, the results indicated that PNI could reflect hepatic functional reserve and nutritional status. Also, the levels of platelets, leukocytes, neutrophils, and lymphocytes in patients with a low PNI were significantly reduced compared to patients with a high PNI, which indicated an inhibited immunity status and severe hypersplenism. These results suggested that PNI might be a valuable prognosis factor for HCC patients, especially in patients with severe liver cirrhosis and hypersplenism. In the current study, approximately 95% of HCC patients had severe hepatic cirrhosis, which is distinctive of HCC patients from China.

Concerning the relationship between prognosis and PNI, the results of the Kaplan–Meier curve and log-rank test indicated that a low preoperative PNI was an independent risk factor for poor OS in HCC patients with HBV infection following US-PMWA. This result was consistent with many previous studies. The multivariate Cox regression models further demonstrated that PNI, tumor diameter and tumor-adjacent organs were independent prognostic factors for OS in HCC patients with HBV infection following US-PMWA. Post-treatment tumor diameter has been previously verified as a prognostic factor in HCC patients (38, 39). However, in the current study, the results indicated that tumor diameter was related to PNI. It is possible that larger tumors might result in reduced hepatic functional reserve, nutritional status, and immune status. However, further analysis is required. The fact that tumors adjacent to organs were considered a risk factor during ablation in HCC patients has been verified by previous studies (40, 41). To avoid the regression dilution bias of multivariate Cox regression models, restricted cubic splines were performed, and the results further indicated the same tendency of a low PNI in patients with poor prognosis. Time-dependent ROC curve analysis is an efficient method to assess the effect of a candidate indicator given the positive events at certain time points. Therefore, time-dependent ROC curve analysis was also performed, which revealed that PNI was closely related to OS of HCC patients with HBV infection after US-PMWA with relatively robust efficiency. Therefore, the preoperative PNI index was verified as a prognostic factor for OS in HCC patients with HBV infection after ablation.

Low PNI is the result of hypoalbuminemia and/or lymphocytopenia. There are several potential mechanisms that might explain how low preoperative PNI could be associated with a poor prognosis. On one hand, HCC is an inflammation-driven cancer in the context of chronic inflammation and liver fibrosis and cirrhosis (42, 43). Albumin is the main component of plasma proteins, and hypoalbuminemia reflects a malnutrition status, and cancer cachexia is caused by a sustained inflammatory response (44). Hypoalbuminemia would damage the cellular and humoral immunity effect, phagocytic effect, and other anti-tumor mechanisms in patients with tumors (45). Impaired synthetic functions of proteins resulting from hepatic cirrhosis need to be considered as an additional determinant for the decrease in serum albumin. On the other hand, lymphocytopenia indicates a weakened anti-tumor immune status (46). Lymphocytes are a crucial component of the immune system, and play a vital role in the biological behavior of HCC, such as tumor initiation, differentiation, proliferation, recurrence and metastasis (44, 47), which affects the tumor microenvironment and prevents tumorigenesis and progression (48). In addition, the depletion of lymphocytes, especially CD4- and CD8-positive T cells, is the key factor for immunosuppression. Lymphocytes, which are present in the tumor microenvironment, seem to be vital for the efficacy of immune surveillance and tumor destruction (49). Therefore, an anti-tumor effect induced by the cellular immunity of T lymphocytes would be reduced during lymphocytopenia (50). Finally, the underlying potential regulatory mechanism of serum albumin and lymphocytes on ablation has not been elucidated and deserves further investigation.

There are some limitations in the present study. Firstly, a retrospective study was conducted in a single center from China. Further prospective studies are needed to confirm the results. The present results also need to be validated in external institutions from different regions. Secondly, the study used 45 as the cut-off of PNI to verify the prognostic value in HCC patients within HBV infection treated with US-PMWA. We should calculate a unique cut-off value and compare it to the data obtained using the cut-off value of 45. Thirdly, laboratory examination information was only collected before ablation, and serial data after ablation might provide more insights into their prognostic values. The change in PNI before and after ablation might also provide promising value. Moreover, due to the inability to obtain accurate data regarding additional treatments following patient discharge, we were unable to analyze the impact of those treatments on the predictive value of PNI. A prospective, multicenter study with a larger sample size is warranted to confirm the results.

## Conclusion

In conclusion, the preoperative PNI was thought to serve as a valuable immune-nutritional indicator for HCC patients with HBV infection in clinical practice. The results of the present study demonstrated that the preoperative PNI represents a convenient, noninvasive, and independent prognostic indicator in HCC patients with HBV infection treated with US-PMWA. This is an easy-to-use prognostic indicator for risk stratification that might contribute to HCC ablation treatment.

## Data Availability

The original contributions presented in the study are included in the article/Supplementary Material, further inquiries can be directed to the corresponding author/s.
